# Low Cycle Fatigue Behavior of Steam Generator Tubes under Axial Loading

**DOI:** 10.3390/ma11101944

**Published:** 2018-10-11

**Authors:** Xing He, Junfeng Chen, Wei Tian, Yuebing Li, Weiya Jin

**Affiliations:** 1 Institute of Process Equipment and Control Engineering, Zhejiang University of Technology, Hangzhou 310032, China; hex@zjut.edu.cn (X.H.); jfchappy@163.com (J.C.); tianwei110924@163.com (W.T.); jinweiya@zjut.edu.cn (W.J.); 2 Engineering Research Center of Process Equipment and Remanufacturing, Ministry of Education, Hangzhou 310032, China

**Keywords:** steam generator tube, fatigue life, Inconel 690, cyclic stress–strain response

## Abstract

Compared with the fatigue properties of the material (Inconel Alloy 690), the real fatigue lives of tubes are more meaningful in the fatigue design and assessment of steam generator (SG) tube bundles. However, it is almost impossible to get a satisfactory result by conducting fatigue tests on the tube directly. A tube with a uniform and thin wall easily fails near the clamping ends under cyclic loading due to the stress concentration. In this research, a set-up for fatigue tests of real tubes is proposed to overcome the stress concentration. With the set-up, low cycle fatigue tests were conducted in accordance with an existing fatigue design curve for Alloy 690. Strain control mode was applied with fully reversed push–pull loading under different strain amplitudes (0.15%, 0.2%, 0.3%, and 0.4%). A favourable result was obtained, and the low cycle fatigue behavior was investigated. The results showed that the fatigue life tested by the real tube was below the strain–life curve of Alloy 690 which was fitted by conventional solid specimens. A cyclic hardening behavior was found by the cyclic stress–strain curve when compared with the monotonic stress–strain curve.

## 1. Introduction

As an important component of nuclear power plants, steam generators (SGs) consist of thousands of tubes that act as heat transfer tubes and the primary barrier between the radioactive and nonradioactive sides. One of the significant safeties of SG tubes issues is the possible rupture [1]. Operational experience shows that SG tubes have been subjected to a number of deterioration and degradation mechanisms [2]. Fatigue damage might be an essential mechanism, including fretting fatigue and environmental fatigue. Flow-induced turbulence and thermal loading due to thermal stratification and thermal striping results in alternating stress on SG tubes. Therefore, the fatigue failure of SG tubes threatens SG reliability.

Although many studies have reported on the fatigue behavior of SG tubes in the last several decades, most researchers focused on the fatigue properties of tube materials rather than real tubes [3,4,5]. Furthermore, most of these researchers investigated the fretting fatigue [6,7,8] and corrosion fatigue of materials to reveal the effect of the water environment [9,10,11,12,13]. However, the fatigue design and assessment of SG tubes usually utilize the fatigue design curve, which is developed based on the fatigue data of specimens from tube materials under normal atmospheric environment and temperature, and consider a series of correction factors. Unfortunately, the fatigue life data of tube materials is very limited [14,15], especially for Alloy 690, which is a substitute material for Alloy 600 due to its superior corrosion resistance. The data compiled including Alloys 600, 690, and 800 are adopted to a fatigue design curve. In addition, the curve is adjusted with correction factors to reflect the effect of water environment or fretting damage [16]. Therefore, it is crucial to obtain much more fatigue life data for the fatigue design of SG tubes. 

It is well-known that the fatigue life might be different for the same material with different cross-sectional shapes. There have been discussions as to the difference in the fatigue behavior of round bar and thin-walled tubular specimens [17,18,19,20]. Tubular specimens are usually adopted to investigate the multiaxial fatigue behavior throughout the literature. In most cases, fatigue lives in hollow-axial tests were marginally lower than those of their companion solid specimens, especially for ductile material. Therefore, it is necessary to investigate the fatigue life of real tubes. 

Most of the fatigue data for Alloy 690 in the open literature was obtained by fatigue tests with round-bar specimens, but little work was based directly on real tubes. SG tubes are typical thin-walled tubes with a small diameter, with which it is impossible to fabricate a bar or plate specimen. The results from round-bar material might not be representative of the real behavior of SG tubes due to the mutually interactive influences of microstructure features, section thickness, and processing variables (e.g., diameter and thickness reduction during cold rolling, etc.). 

Conventional tubular specimens are manufactured as a hollow barbell shape, whose external profile is similar to round bar specimens. These specimens with barbell shape effectively enhance the stress level on the gauge in comparison with the stress concentration on the ends due to the clamp. Therefore, they tend to clamp and achieve failure in gauge length. However, it is difficult to fabricate a standard specimen with a hollow barbell shape for an SG tube with a uniform and thin wall. Generally, an SG tube is a typical thin-walled tube, with diameter of 17.46 mm and thickness of 1.04 mm. To obtain the fatigue properties using SG tube, the clamping issue needs to be solved. 

Clamping might distort the cross section of the tubular specimen. Several clamping methods have been proposed. Weiss and Stickler [21] proposed a specially designed adapter tip to perform ultrasonic fatigue testing of thin-walled stainless steel tubes, where the connection between tube sample and adapter tip was made by silver solder [22]. With a special clamping system, Yoon et al. determined the low fatigue resistance of 429EM stainless steel tube [23]. Lambertsen et al. designed a bushing system that consists of a solid round bar with large gripping diameter and a part that fits inside the tube [24]. Glue is infused into the space between the tube bushes. For the high cycle fatigue regime of thin-walled tubes [25], tube samples featuring holes in their midsection were specially prepared for ultrasonic resonance fatigue testing.

The main objective of this work is to present the fatigue properties of SG tubes. In this work, fatigue tests are carried out on tube specimens cut from real SG tubes. A locally thinned tube specimen and auxiliary clamping technology are proposed. Fatigue lives are tested under reversal strain loading. The cyclic stress–strain response and fracture morphologies of the fatigue-tested specimens are analysed. The fatigue lives of these tube specimens are compared with solid specimens. 

## 2. Materials and Experimental Procedure

### 2.1. Materials

**Figure 1:**
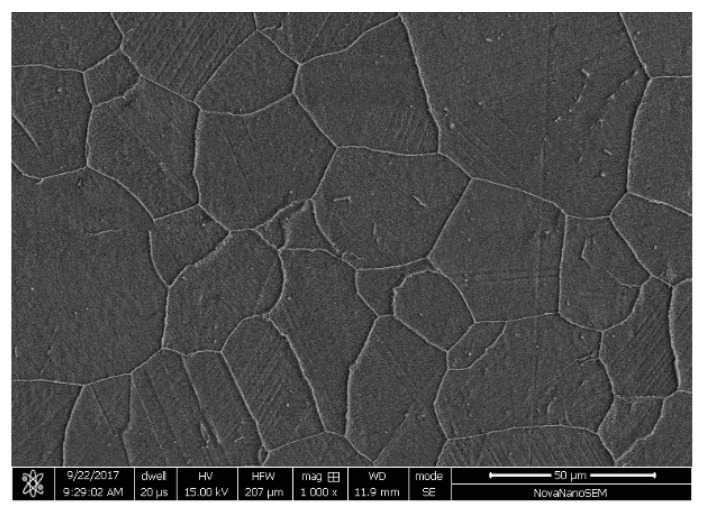
SEM morphology of microstructure.

Chinese SG tube made of Inconel Alloy 690 was used in the present work. The composition of Inconel Alloy 690 is listed in Table 1. The tubes used in this work were cold drawn from solid bar. The tube consisted of near equiaxed grains and discrete carbides M23C6 with average grain size of about 30 μm (Figure 1). 

**Figure 2:**
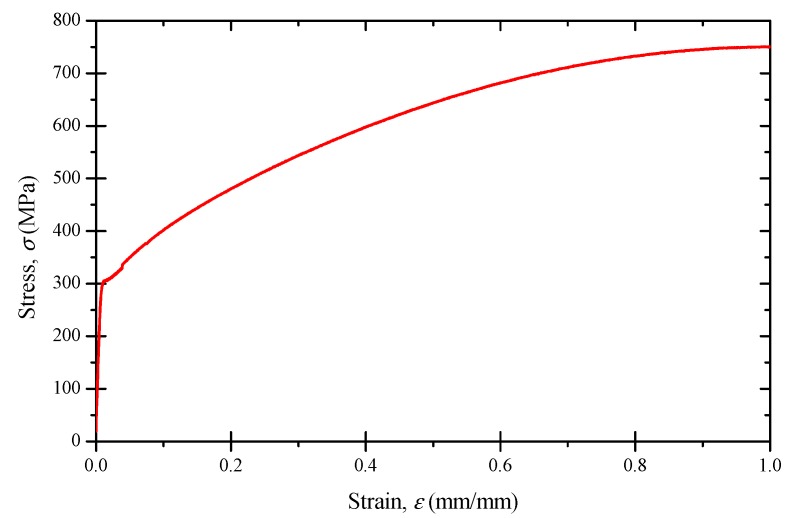
Stress–strain curve of Inconel 690 tube.

A standard procedure according to ASTM E8 [26] was used to perform tension testing by using full-size small tube specimens. The tensile properties of the tube were tested as shown in Figure 2, and results are listed in Table 2. It can be seen that the tube exhibited good ductile property. 

### 2.2. Specimen and Experimental Procedure

**Figure 3:**
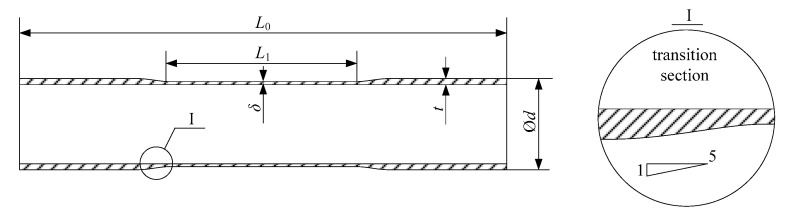
The thinned tubular specimen of Inconel 690.

Because of the very small diameter and thickness, it is difficult to produce a standard fatigue specimen according to ASTM E606 [28]. Similar to the tubular specimen adopted to study multiaxial fatigue behavior, a locally thinned tube is proposed as shown in Figure 3. The middle part of the tube (*L*_1_) was worn off along the thickness by precision grinding. The length of the worn off section which was used for the extensometer to measure deformation was set to be 35 mm, equivalent to at least twice the tube diameter. The whole length of the tubular specimen *L*_0_ was 120 mm. Two values of the remaining wall thickness (*δ* = 0.5 mm and 0.8 mm) were considered with strain loading to increase the stress. Meanwhile, a smooth transition with falling gradient under 1/5 was manufactured to prevent the failure from occurring at the transition section.

**Figure 4:**
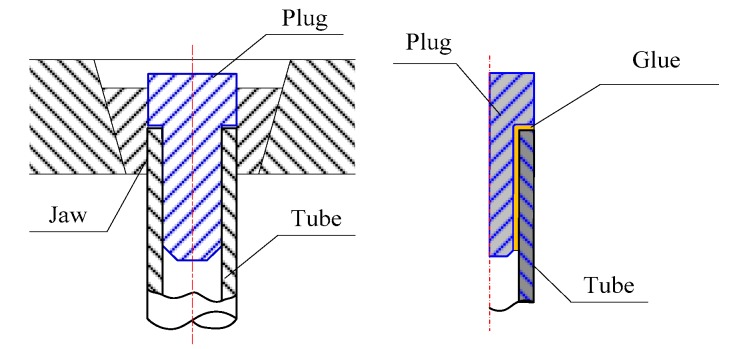
Clamping models for tube.

According to ASTM standard E606, snug-fitting metal plugs should be inserted far enough into the ends of such tubular specimens to let the testing machine jaws grip the specimens properly, as shown in Figure 4. The gap between the plug and tube was about 0.03 mm, which was infused with strong glue. 

The fatigue tests of SG tubes were conducted at room temperature using an Instron 8850 servo hydraulic test system (Instron, Norwood, MA, USA). Constant amplitude strain-controlled uni-axial tests were carried out under fully reversed push–pull loading with a frequency of 10 Hz in sinusoidal wave form. Four different values of strain amplitudes (*ε*_a_ = 0.15%, 0.20%, 0.30%, and 0.40%) were considered. During the tests, we first used three tube specimens with remaining thickness of 0.8 mm for strain amplitude *ε*_a_ = 0.40%, and obtained a favourable result. However, the specimens with remaining thickness of 0.8 mm were not suitable for lower strain amplitudes. Then, the specimens with remaining thickness of 0.5 mm were adopted for the other strain amplitudes. After fatigue tests, the specimens were sectioned. The fracture surface and sectioned area were examined using a field-emission scanning electron microscope (FE-SEM, Quanta 200F, FEI, Hillsboro, OR, USA).

## 3. Results and Discussion

### 3.1. Validity of the Prepared Tube Specimen

**Figure 5:**
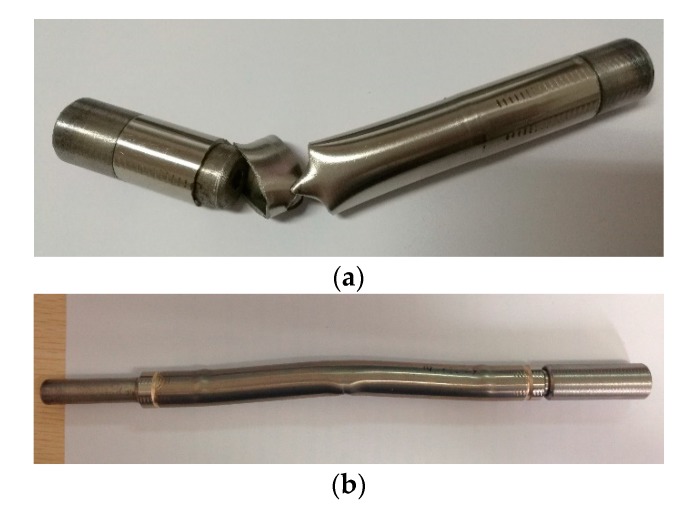
Typical failures of as-received tube: (**a**) rupture near the clip position; (**b**) buckling.

Before the set-up of the clamping approach with locally thinned tube, the fatigue life of as-received tube was also tested. Having tried a set of clamping models, several unexpected results occurred, such as rupture near the clamping position (as shown in Figure 5a) and buckling (as shown in Figure 5b). As the real tubes have uniform thickness, the stress level for the whole tube under axial loading is well-distributed. However, the clamping force will result in certain local damage on the tube ends, and fatigue failure easily occurs in the clamping area. Further, a long tube specimen buckles easily.

**Figure 6:**
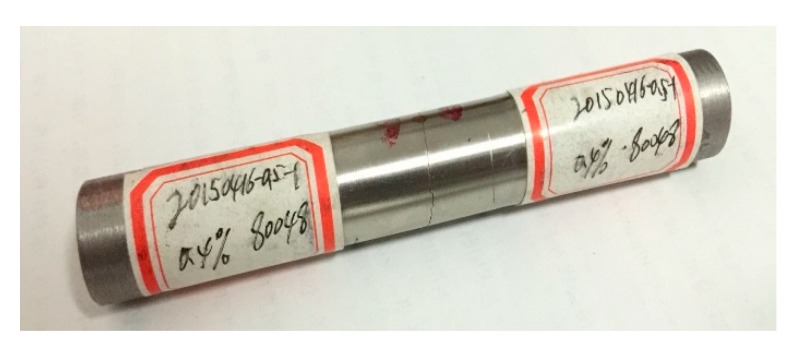
Typical failure of thinned tube.

To prevent failure from the clamping ends, the locally thinned tube specimens were designed as described in Section 2.2. Figure 6 shows the rupture pattern of a locally thinned tube specimen, where the tube ruptured in the thinned area. It can be seen that there was an apparent crack on the external surface perpendicular to the loading axis, which indicates that fatigue crack initiated in the gauge length. The appearance validates the specimens with locally thinned tube very well. The test results can reflect the realistic fatigue life of SG tube. The set-up of the clamping approach with locally thinned tube specimens can significantly reduce the effect of stress concentration due to the holding device.

### 3.2. Fatigue Life

**Figure 7:**
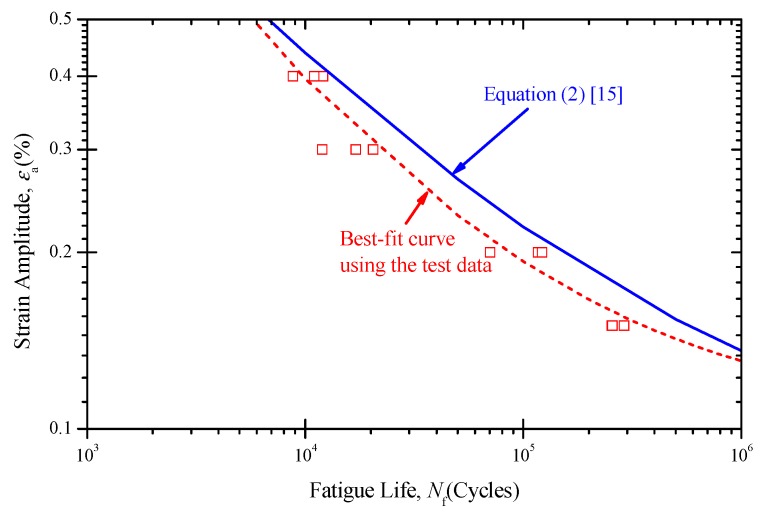
Test data for steam generator (SG) tubes.

A total of 12 test results under different strain amplitudes (0.15%, 0.2%, 0.3% and 0.4%) were obtained, as shown in Figure 7. Fatigue life was defined as the number of cycles for tensile stress to drop 25% from its peak value. Based on the test data, a strain–life cure was fitted as Equation (1) in terms of the Langer equation [29]. For comparison, the best-fit curve for nickel base alloys [15,16] described by Equation (2) was also plotted, which was used to develop the existing fatigue design curve for Alloy 690. It should be noted that the limited fatigue data used in fitting the curve were based on strain-controlled tests of small polished solid specimens at room temperature surrounded by air. In addition, three types of nickel base alloy widely used in SG tubes were compiled, including Alloys 600, 690, and 800.

(1) $$\varepsilon_{a}=32.17N_{f}^{-0.5101}+0.1026$$

(2) $$\varepsilon_{a}=14.967N_{f}^{-0.4053}+0.0805$$

It can be seen that all tested fatigue lives were located below the best-fit curve. This might be for several reasons, such as the effects of raw material, heat treatment, processing technology, surface finish, etc. However, fatigue tests in this work used SG tubes rather than the material itself. In the design of SG tubes, a fatigue design curve based on tubes is much more accurate than one based on materials. 

For the locally thinned tube specimen, only the surface roughness was changed in comparison with SG tubes. The surface roughness was investigated with this in mind. Table 3 shows the average surface roughness of as-received and thinned tube specimens. As observed in Table 3, the as-received tube exhibited a relatively low roughness on both internal and external surfaces. However, the surface roughness on the external surface of the thinned tube specimen was about five times that of the as-received tube. This might be one reason why all tested fatigue lives were located below the best-fit curve. In general, fatigue lives decreased as surface roughness increased. Surface damage and alteration is one reason for the decrease of fatigue life. In the following work, a much finer surface will be imparted to determine the influence of specific surface conditions on fatigue life.

### 3.3. Cyclic Stress–Strain Response

**Figure 8:**
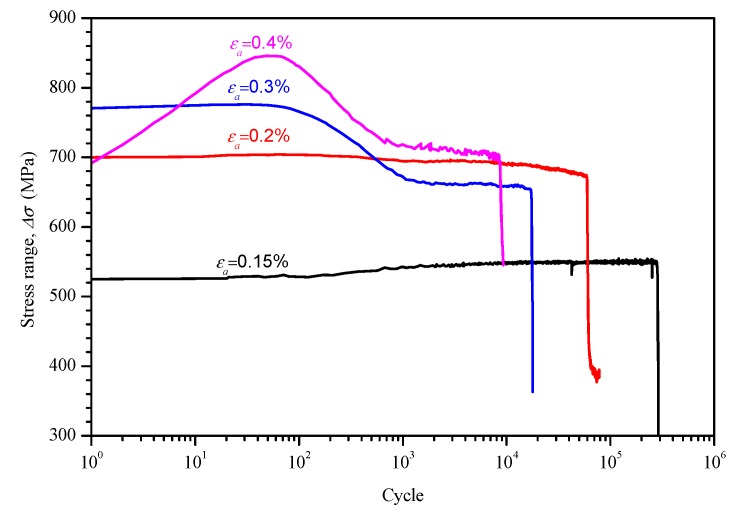
Cyclic stress range with number of cycles.

Figure 8 shows the stress ranges varied with cycles for different strain amplitudes. At a strain amplitude of 0.4%, the tube specimen exhibited a normal hardening and softening response followed by a stability process until the onset of final load drop. The present finding is in accordance with the reported behavior of a hot-extruded Alloy 690 material [4], which is revealed by the dislocation annihilation rate. At a strain amplitude of 0.3%, there was no obvious hardening response, except at short lives (about 100 cycles in the beginning). At strain amplitudes lower than 0.3%, only a gradual hardening was observable until the onset of final load drop. This indicates that the dislocation annihilation rate strongly depended upon strain amplitude. 

**Figure 9:**
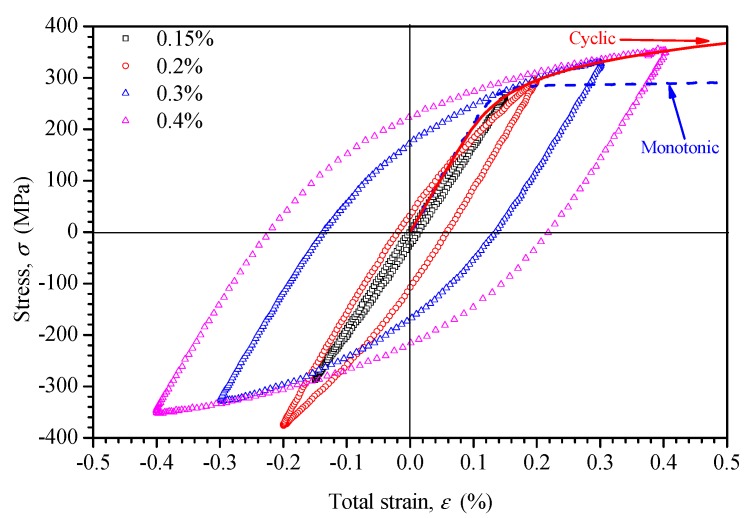
Stress–strain hysteresis loops and the fitted cyclic stress–strain curve by the test data.

In general, the hysteresis loops stabilized approximately at the half-life of the specimens during testing. The cyclic stress–strain curve was determined by connecting the tips of the half-life hysteresis loops of the fatigue-tested specimens for different strain amplitudes, as shown in Figure 9. In comparison with the monotonic stress–strain curve, the cyclic stress strain curve showed a cyclic hardening behavior, especially for large strain amplitudes. 

The cyclic stress–strain curve can be represented by the following power law relationship [30]:

(3) $$\frac{\Delta \varepsilon }{2}=\frac{\Delta \sigma }{2E}+{\left(\frac{\Delta \sigma }{2K^{\prime }}\right)}^{1/n^{\prime }},$$

where $K^{\prime }$ is the cyclic strength coefficient, $n^{\prime }$ is the cyclic strain-hardening exponent, Δσ/2 is the cyclic stress amplitude, Δε/2 is the cyclic strain amplitude, and *E* is the Young’s modulus. The cyclic properties were determined from the data, with $K^{\prime }=424.92\;\mathrm{MPa}$ and $n^{\prime }=0.129$ in Equation (3) for strain amplitudes lower than 0.4%.

### 3.4. Fractography Analysis

**Figure 10:**
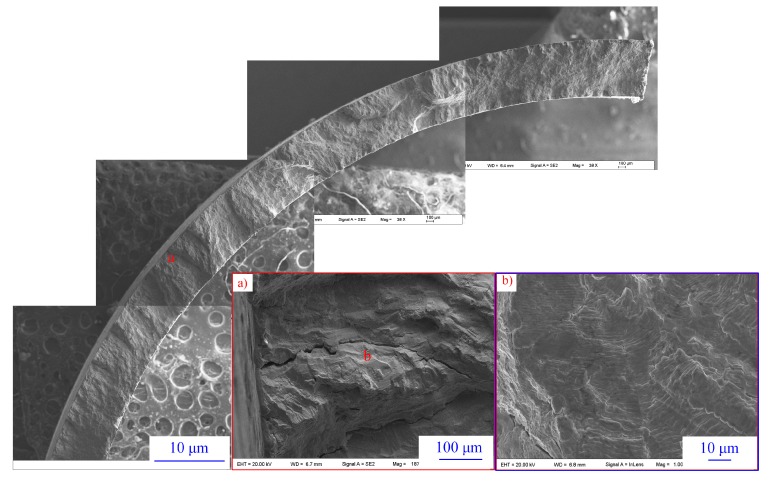
Typical morphologies of surface cracks on SG tube specimens after fatigue tests.

Fractography analysis was carried out on these specimens after fatigue tests. River lines with facets and striations could be easily observed in the near-threshold fatigue regime, as shown in Figure 10. The apparent site of crack initiation could be identified as the point from where the river lines originated. On all specimens, the crack initiation site appeared to be at or adjacent to the surface of the tube. In addition, well-developed striations normal to the crack growth direction during the propagation stage were observed on the fracture surfaces of the specimens.

## 4. Conclusions

The low cycle fatigue behavior of SG tubes under axial loading was investigated using locally thinned tube specimens cut from real SG tubes. Fatigue tests were conducted under fully reversed push–pull loading at different strain amplitudes (0.15%, 0.2%, 0.3%, and 0.4%). Based on the results and analysis presented in the paper, the following conclusions can be made.

(1) Apparent cracks on external surface of the thinned domain could be found following fatigue tests, perpendicular to the loading axis. Failure dominated by stress concentration due to the holding device could be avoided with the proposed test scheme. The test results showed the real fatigue life of SG tube. 

(2) The fatigue lives of real tubes were obtained in strain control mode. Compared with the best-fit curve on the material fatigue data, real tubes had lower fatigue life under the same strain amplitude. However, rough surface appeared during thinning, which may reduce the fatigue life.

(3) The results of the cyclic stress–strain response investigation showed that the response strongly depended upon strain amplitude. At large strain amplitude, the tubes exhibited a hardening and softening response after a stability process. However, there was no obvious hardening response for low strain amplitude. A cyclic stress–strain curve was also fitted.

(4) On all specimens, the crack initiation site appeared to be at or close to the surface of the tube. River lines with facets and striations could be easily observed in the near-threshold fatigue regime.

## Figures and Tables

**Table 1:** Chemical compositions of Inconel 690 (wt %).

Ni	Cr	Fe	C	Mn	Si	Cu	S
58.0 min.	27.0–31.0	7.0–11.0	0.05 max.	0.50 max.	0.50 max.	0.5 max.	0.015 max.

**Table 2:** Tensile properties of Inconel 690.

Item	Young’s Modulus (MPa)	Yield Strength (MPa)	Tensile Strength (MPa)
This work	2.12 × 10^5^	286	702
ASME SB-167 [27]	--	240	586

**Table 3:** The average surface roughness of as-received and thinned tube specimens.

Site	Internal Surface	External Surface	
		As-Received Tube	Thinned Tube
R_a_ (μm)	0.135	0.070	0.342
